# Fifteen Minutes per Day Keeps the Violence Away: a Crossover Randomised Controlled Trial on the Impact of Foot Patrols on Serious Violence in Large Hot Spot Areas

**DOI:** 10.1007/s41887-021-00066-3

**Published:** 2021-08-17

**Authors:** Matthew Bland, Michelle Leggetter, David Cestaro, Jacqueline Sebire

**Affiliations:** 1 Institute of Criminology, Sidgwick Avenue, Cambridge, CB39DA UK; 2 Police Headquarters, Wobourn Road, Kemptson, Bedfordshire, MK43 9AX UK

**Keywords:** Hot spots, Knife crime, Serious youth violence, Violent crime, Foot patrol, Deterrence, Residual deterrence, GPS tracking, Crime harm index

## Abstract

**Research Question:**

Did a 15-min patrol delivery over 1 day reduce serious violent crime in large hot spots (mean size = 2 km × 2 km), without displacing such crimes to nearby areas?

**Data:**

We tracked daily official crime reports in a sample of 21 high-crime Bedfordshire (UK) Lower-layer Super Output areas (LSOAs). We measured time spent by two-person police foot patrols in those areas with daily GPS data from handheld devices given to officers working on overtime. We also counted proactively initiated arrests.

**Methods:**

We used a crossover randomised controlled trial on the 21 “hot spot” LSOAs, each of which was randomly assigned daily to be either in a treatment condition of 15-min of patrol (as one of seven each day) or a control condition of no patrol (as one of 14 each day) for each of 90 days. We used an intention-to-treat framework to analyse the impact of patrols on the outcome measures overall, on consecutive days of assignment to the same condition, and in 100-m ‘buffer’ zones around each hot spot.

**Findings:**

We found that on treatment days the hot spots had 44% lower Cambridge crime harm index scores from serious violence than on control days, as well as 40% fewer incidents across all public crimes against personal victims. Statistically significant differences in lower prevalence, counts and harm of both non-domestic violent crime and robbery and other non-domestic crimes against personal victims were also found. We found no evidence of either displacement of serious crime into a 100-m buffer zone, nor any evidence of residual deterrence on no-patrol days following patrol days. We did find evidence of a cumulative effect: the largest differences in crime harm on control days were found in treatment days that came after 3 days of consecutive patrol in the same LSOA.

**Conclusions:**

Even minimal amounts of foot patrol can prevent serious violent crime across a large area, and repeated patrols over several days help even more. Our findings suggest that, to reduce both violent and other forms of crime, uniformed officers need to patrol hot spots for short amounts of times on consecutive days.

## Introduction

Hot spot policing, among all crime prevention strategies, has what may be the largest body of research evidence supporting its cost-effectiveness (Braga et al., 2019). Yet it can also claim the largest gap between research and practice. In the UK, for example, despite repeated demonstrations of the effectiveness of small doses of foot patrol in crime hot spots (Ariel et al., 2016, 2020), we know of no police agency that has implemented a comprehensive, force-wide patrol strategy to target hot spots of violence, with precision and close tracking, on a daily basis.

Such a strategy has been especially viable after repeated investments of extra funding for policing against serious violence, as of 2021 worth over £100 million in total (Home Office, 2019). Increasingly, the UK Home Office has encouraged high visibility policing in ‘hot spots’. This study is a direct result of that funding strategy. In this paper, we present the results of ‘Operation Rowan’, a crossover randomised controlled trial run by Bedfordshire Police.

Op Rowan’s primary purpose was to test the efficacy of foot patrols on violent crime in hot spots, both in terms of overall effect and to optimise patrolling patterns. The experiment was conceived to seek conclusions about ‘how much is enough’ patrol to reduce serious violence. The study design was informed by the finding that one patrol every 3 to 4 days was able to reduce crime harm substantially, as first reported in Perth, Australia (Barnes et al., 2020). For Bedfordshire, Op Rowan was designed to measure the specific effects of foot patrol on the reduction of serious violent crimes. It was also designed to use a very large unit of analysis, larger than any previously reported field experiment: Lower-layer Super Output Areas (LSOAs) with a mean of 2000 m^2^. By comparison, the Perth experiment tested patrol effects in areas of 200 m^2^, or 90% smaller than in our study.

In their systematic review of 78 field tests, Braga et al. (2019) concluded that the mean effect size of hot spot policing on violent crime is lower than its effects on property crime, disorder or drug crime (Table 2, p. 300). Yet this analysis does not include a randomised test of patrols in large areas.

Our study is the first randomised controlled trial (RCT) in the UK to test the hypothesis that short periods of foot patrols will reduce violent crime in large LSOAs. Yet like previous studies, our framework is founded on two specific cornerstones of criminology: (1) that crime is *concentrated* in certain places and (2) that it can be reduced via means of *general deterrence*.

The ‘law of crime concentration’ (Weisburd, 2015) is founded on consistent evidence that crimes occur repeatedly in a small proportion of places (e.g. see Andresen & Malleson, 2011; Farrell, 2015; Sherman et al., 1989; Weisburd & Amram, 2014; Weisburd et al., 2012). As Weisburd (2015) reports, a majority of place-based studies generally find that most crime takes place in fewer than 10% of total locations. This is the fundamental premise of the first experiment in ‘hot spot policing’ (Sherman & Weisburd, 1995) and all subsequent tests: that targeting these hot spot locations with additional interventions can reduce crime.

The appropriate size of these ‘hot spots’ is a debate that runs consistently through the existing body of studies on crime concentration and hot spot policing. The general trend of advocacy is towards smaller being better—‘microgeographic units’ as Weisburd (2015, p. 137) labels them. The size and shapes of these units in the literature are variable, including individual addresses (Eck et al., 2000; Pierce et al., 1988; Sherman et al., 1989), street segments (Curman et al., 2015; Weisburd & Mazerolle, 2000; Weisburd et al., 2004) and grid shapes (Ariel et al., 2016; Williams & Coupe, 2017). Yet it remains possible that hot spot patrols can work in places both very large and very small. The present test may deploy patrols to the largest areas yet tested.

The second criminological foundation of direct relevance to our experiment is general deterrence theory: the claim that the threat or application of sanctions for criminal behaviour has an effect on the rate of crime in any given population (Beccaria, 1763; Zimring & Hawkins, 1973). The first component of this theory is that the act of crime is a choice by the offender (see Clarke & Felson, 1993). The practicality of deterrence theory in action, in which a potential offender makes a determination about the risk of punishment linked to their potential offence, is also associated to ‘prospect theory’ (Kahneman & Tversky, 1979).

General deterrence now has a conceptually rich body of research with several variants, including ‘local’ deterrence, in which a deterrent effect manifests in a specific geography owing to a locally targeted intervention such as a patrol (Sherman et al., 2014); ‘regional’ deterrence as described by the theoretical framework for diffusion of benefits (Clarke & Weisburd, 1994); ‘initial’ deterrence, described by Sherman (1990) as crime reduction in the immediate aftermath of an intervention; and ‘residual’ deterrence (Sherman, 1990) described as the carryover effects of an intervention into time periods after the intervention has ceased or police have left the location.

Nagin (1998) illustrates residual deterrence through the perspective of ‘ambiguity aversion’, which ties directly to prospect theory. In terms of patrols, ‘ambiguity aversion’ might work like this: potential offenders witness a patrol in passing, and this informs their judgement about the risk of being caught and punished for committing crime. The recent memory of seeing the patrol increases the perceived risk even after the patrol has ended, because of the uncertainty felt by the potential offender. This is an example of the role of perceptions of risk being critical to the mechanisms of deterrence as well as the *actual* certainty, severity and celerity of punishment. In itself, this element may attract its own label of ‘perceptual’ deterrence (Barnum et al., 2021), but it should be generally recognised that these labels are not necessarily mutually exclusive. The mechanisms through which deterrence works in practice are mostly unknown, despite the large body of theoretical and empirical research that concerns the theory.

In practice, a large array of factors interacts with the mechanism of deterrence by police patrol, such as capable guardianship, individual choice processes (Paternoster, 1987), indirect and direct shaming effects (Sherman, 1993; Stafford & Warr, 1993) and individual levels of self-control (Nagin & Paternoster, 1993; Piquero & Tibbets, 1996). We do not attempt to distinguish these elements in our research design. Our primary concern is the deterrent effect of foot patrols on violent crimes, by whatever causal mechanisms may affect the nature and frequency of the crimes we are measuring.

Our approach emphasises the *measurement* of police presence over the *mechanisms* by which it may prevent crime. A key part of the hot spot policing methodology in the past decade has been the recording of the amount of patrol actually delivered in each place on each day (often referred to as patrol dosage—see Sherman et al., 2014). GPS tracking has been successfully deployed in UK experiments in Peterborough (Ariel et al., 2016), Birmingham (Williams & Coupe, 2017), as well as in Sacramento, CA and Perth, Australia (Barnes et al., 2020).

Our predominant interest is in detecting the presence of residual deterrence (Sherman, 1990), especially on the days when patrols are provided for just 15 min but with residual effects that may last all day or longer. Our design was informed by the *residual deterrence* reported in Koper’s (1995) analysis of the ‘Koper Curve’ in the original Minneapolis hot spot patrol experiment, which identified 10–15-min patrols as the optimal length of time for maximising crime reduction for 30 min after police left. As Koper found, most of the prevention of crime in that experiment occurred when the police were not present, or 85% of the time. Similarly, in the Ariel et al. (2020) London Underground platform patrol experiment, 97% of the crime prevention effect was the carryover residual deterrent effect when officers were not patrolling. None of these residual effects, however, were as large as the multi-day post-patrol effects in the Perth (AU) “Sweet spots” experiment.

### Building on the ‘Sweet Spots’ Experiment

Barnes and colleagues’ (Barnes et al., 2020) ‘sweet spots’ study of minimising patrol time took place in Perth, Australia between August 2017 and April 2018. Barnes and his team identified 15 hot spots, each 200 m^2^ in size, all of which were within high population areas but relatively well spread out across the city (most being more than 1 km apart). For each of the experiment’s 248 days, the 15 hot spots were randomly assigned to three conditions, including vehicle patrol, bicycle patrol or ‘business as usual’, the latter being the control condition. No instructions were given to officers about what to do on their patrols other than to visit the designated hot spot twice, with each visit being for 20 min. Officers were issued with GPS enabled phones to track their locations.

In shuffling the treatment condition of each hot spot each day, the “Sweet Spots” design made each individual hot spot its own control unit as well as its own treatment unit. The unit of analysis became location-days, and the analysis measured average outcomes across days in both conditions. “Sweet Spots” experimental location-days recorded more patrol than control location-days in terms of prevalence, frequency and average minutes of *patrol* (Barnes et al., 2020, Table 1). It reported that the prevalence, mean volume and mean harm of *crimes* on treatment location-days was lower than on control equivalents (though prevalence was not found to be statistically significant). In all cases, the effect sizes were small. While the experiment reported little impact of consecutive days’ patrol, it identified a ‘collapse’ in deterrence effects after 4 days with no patrol, after which a fivefold increase in crime harm was observed.

**Table 1:** Sensitivity and specificity analysis of 3-month rolling risk thresholds

A. LSOA risk of serious youth violence in the next 30 days	B. Number of LSOAs targeted	C. Number of LSOAs not targeted	D. Percent of LSOAs targeted which have a crime in 30 days (sensitivity)	E. Percent of LSOAs not targeted which have a **no** crime in 30 days (specificity)	F. Max percent of crime prevented
90%	3	349	100%	89%	12%
35%	10	342	70%	90%	21%
20%	30	322	36%	91%	31%
15%	93	259	21%	92%	49%

The major implication from Sweet Spots is crime reduction with ‘minimalist’ patrolling. Barnes and colleagues estimated an annualised reduction of more than 40% volume and 80% harm between treatment and control days, which is substantial. Yet one test alone cannot establish whether similar effects would be achieved elsewhere, and under different conditions. One key issue is not just how little patrol is provided, but for how large an area. In this test, we apply a crossover design for patrolling areas in Bedfordshire that are ten times larger than the hot spots in the ‘Sweet Spots’ test.

## Research Questions

Under special funding from the Home Office, the primary outcome measure in the Bedfordshire RCT was serious violent crime. Our major obstacle in tracking this objective was statistical power. Consequently, we adjusted our outcome measures on the basis that the intervention has been shown to work on multiple types of crime (Braga et al., 2019).

As with the “Sweet Spots” experiment, we were interested in not just the average treatment effect of patrol versus no patrol, but also residual deterrence and displacement. We formulated the following research questions:

1. What is the average effect of targeted foot patrols on the prevalence, volume and harm of:
  - violent crimes (including robberies) that are not domestic abuse.
  - all other non-domestic abuse crimes against victims.
  - all non-domestic abuse crimes that are discovered by police proactivity.
  - anti-social behaviour events.^1^
2. What is an optimal pattern of repeated days of patrol for reducing prevalence, volume and harm of the same categories of crime and incident (a–d)?
3. To what extent do targeted foot patrols lead to displacement into local adjoining land of the same four categories of crime and incidents (a–d above)?

## Data

### Targeting Within All Possible Units of Analysis

To define and select the experimental hot spots, we operated on the ‘power few’ principle, in which we judged the maximum likelihood of detecting an effect lay in the highest harm locations. This also made for the most pragmatic operational strategy for Bedfordshire in terms of targeting serious youth violence. To this end, we extracted all crimes designated by Bedfordshire analysts as ‘serious youth violence’ and mapped them by Lower-layer Super Output Area^2^ (LSOA). Replicating Massey et al. (2019) conditional probability methodology, we calculated the monthly risk of such crimes in each LSOA on a rolling basis, given each area’s incidence of the crime in the previous 90 days (see Table 1, which shows a breakdown of the conditional probability of an LSOA having a serious youth violent crime in the following 30 days and the potential outcomes from targeting LSOAs at each threshold of probability). Table 1 can be read as follows:

- Each row represents a different threshold of probability that a serious youth violent crime would occur in an LSOA in the next 30 days.
- In the top row, this threshold is 90% (A).
- There were three LSOAs which had a 90% probability of another serious youth violent crime in the 30 days after the last (B).
- Targeting three LSOAs for a proactive intervention would mean not targeting the other 352 LSOAs that there are in the county (C).
- Of the three that were targeted, all of them would go on to have a serious youth violent crime (D).
- And of those 349 not targeted, the vast majority (89%) would not have a serious youth violent crime (E).
- In targeting the three LSOAs at this threshold, we have a chance of preventing 12% of the total serious youth violence in Bedfordshire (F).

The key takeaway of this analysis was that by targeting just 30 (8.5%) of Bedfordshire’s 352 LSOAs, we could address over 30% of the volume of serious crime, thus establishing some basis of the ‘law of crime concentration’ in action. Furthermore, we established that the LSOAs with the highest conditional probability did not change greatly from month to month. We further refined the analysis with the addition of crime harm weightings, using the Cambridge Crime Harm Index (CCHI, see Sherman et al., 2016) and established a four-tier classification of LSOAs based on how frequently they would be targeted at a 30% conditional probability threshold (as shown in Table 2).

**Table 2:** Classifications of LSOAs based on conditional probability of serious youth violence over 1 year

Classification	Number of LSOAs in this category	Proportion of overall crime count	Proportion of overall crime harm	True positive rate
Chronic	8	24.1%	26.8%	63.1%
Frequent	20	17.7%	13.8%	23.1%
Occasional	58	26.6%	24.1%	10.5%
Rare	266	31.6%	35.3%	0.4%

Chronic = 20% or higher conditional probability of serious youth violence in the next 30 days, in 66% or more of forecasts (*n* = 20); frequent = 50% or more of forecasts ≥ 20%; occasional = 33% or more of forecasts; rare = less than 33% of forecasts.

Table 2 further shows that a small proportion of LSOAs in Bedfordshire consistently experience serious violent events. By targeting just these 28 (chronic and frequent) LSOAs, Bedfordshire Police might address up to 40% of the volume and harm of these crimes.

### Patrolling on Foot

**Fig. 1:**
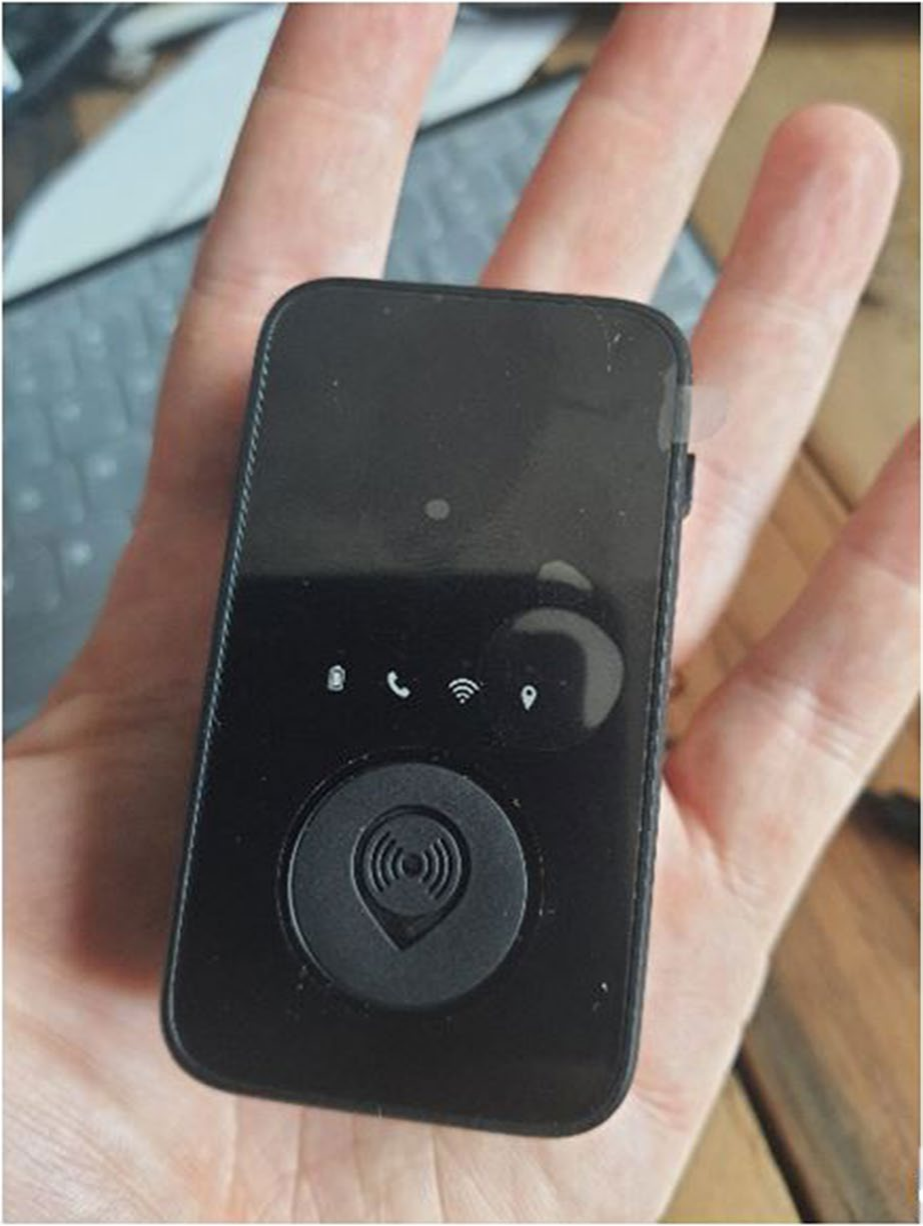
Handheld GPS device for tracking foot patrols

Our intervention was patrolling on foot. Officers were assigned to conduct a minimum of 15-min patrol in each hot spot area which was monitored by use of a handheld GPS device (see Fig. 1). A dozen devices were circulated to officers in Bedfordshire and kept in local police stations. Each day, officers assigned to Operation Rowan duty would collect a device, turn it on and keep it about their person during their shift.

As in the Perth ‘Sweet Spots’ RCT, we were unable to monitor the activities of all officers on duty at any given time. The extent to which we did observe general patrol duties outside of Operation Rowan tasks gives us reassurance that the hot spots (and other areas) were not being routinely exposed to 15 min or more of foot patrol. Capacity issues meant that officers assigned to community policing teams were highly unlikely to deliver foot patrols in targeted hot spots on control days, since their time was taken up with reactive tasks. Instead, a ‘proactive’ pool of officers and PCSOs (drawn from both response and community policing teams) was assigned to complete the foot patrols in those places as a priority on each treatment-assigned day.

The GPS devices enabled our team to track patrols in real time, through a GIS dashboard. The devices were set to ‘ping’ at 20-s intervals, meaning we could assess the speed of travel and location with a high level of precision. Five consecutive minute movement at a rate of one to six miles per hour was set as the threshold for determining ‘foot patrol’. In practice, each use of a GPS device was scrutinised individually. That level of intensity would probably not be feasible in a wider roll-out of the intervention, but it greatly boosted the reliability of measurement of patrol dosage in the experimental setting.

The GIS dashboard was used to compile a weekly tracking report which was provided to senior officers for feedback regarding treatment compliance. In turn, this fed directly into daily management meetings.

### Dependent Variables: Crime, Harm & Disorder

The source of crime data to measure outcomes was Bedfordshire Police’s records management system, Athena. In collating outcome crimes, we extracted all crime records which did not have a domestic ‘flag’ (used to indicate they were domestic abuse). Our intention was to analyse only crimes that occurred in a public space. With no reliable indicator of this dimension, we opted simply to exclude ‘domestic abuse’ crimes on the basis that the majority of these occur in private spaces. We were also able to exclude a number of further crime categories on the basis that the description of the crime indicated that the crime did not take place in a location where police could see it. These included malicious communications crimes, blackmail, taking of indecent images of children and the facilitation of travel for exploitative purposes. We also excluded all crimes relating to the HMP Bedford prison, which took place within the contained environment of the prison grounds.^3^

The experiment ran from 9 November 2020 to 6 February 2021; any crimes committed outside this period were excluded from the final analysis. As we needed to assign crimes to location-days (see next subsection) with a high degree of accuracy, we also excluded any crimes that could not be identified as happening within a 24-h window. Any crimes that were reported within a 24-h window but overlapped two separate days were assigned to a particular date based on the balance of probabilities (i.e. if a crime occurred between 22:00 on day 1 and 01:00 on day 2, we would assign this as day 1 because it had two possible hours for this day compared to only one possible hour for the second day).

We categorised eligible crimes in the following way:

- Violent crimes and robbery—which consisted of assault with and without injury, assaults on police and other emergency workers, harassment, threats to kill and both personal and commercial robberies.
- Other crimes against personal victims—including sexual offences, thefts, burglaries, criminal damage and arson.
- Proactively detected crimes—including drugs offences, possession of weapons, public order and miscellaneous crimes against the state.

Categorisations (b) and (c) take their lead from the classifications of crime suggested by the Cambridge Consensus Statement (Sherman & Cambridge University associates, 2020) recommending a more nuanced approach to crime statistics. The main pillar of this statement is that crimes vary widely in their origin, and assessing performance (or in our case, impact) by grouping crime types of different origin together creates great difficulty of interpretation. For example—drug crimes are most often the product of proactive police activity (such as a warrant or a stop-search) rather than the result of a witness or victim report. We cannot direct foot patrols not to intervene in situations where such crimes may be uncovered because they may dilute the effects of the patrol on experimental measurements, but we can separate such crimes and adjust our expectations accordingly. Thus, we hypothesise that we will see a reduction in crime in measures (a) and (b) above, but not necessarily in (c).

Our measurements of crime harm are taken directly from the latest version of the Cambridge Crime Harm Index (see www.cambridge-ebp.co.uk/crime-harm-index). We made no distinction in our analyses for knife or other forms of weapon crime, nor the age of the suspect or victim. Anti-social behaviour data were taken from the force command and control system, STORM. The same time-window criteria were applied to these data as were applied to the crime data.

### Experimental Design

We selected the 21 highest frequency LSOAs from the 28 ‘Chronic’ and ‘Frequent’ classifications shown in Table 2, setting the bottom seven aside due to patrol resource limitations. Using Microsoft Excel, we randomly assigned each LSOA to two conditions (patrol and no patrol) for 90 days. The randomisation was undertaken at the outset of the experiment and ‘drip-fed’ to the on-site Project Manager on a week-by-week basis. Supervising officers were issued with their instructions for the week ahead by Friday of each week. While this approach was not a fully blinded method, we had little indication of any effort to confound random assignment (see Sherman’s [1986] description of intentional violations of control areas in the Kansas City Patrol Experiment).

Our unit of analysis was location-day, with 21 locations × 90 days = 1890 units in total. For each day, seven hot spots were assigned to receive at least 15 min of foot patrol by a uniformed officer during the 10-h late shift (14:00–00:00). We did not specify what type of officer should undertake how many or what activities. We only specified that the patrol should be conducted on foot and that officers should seek to engage with members of the public. Officers were aware they were being monitored as they were issued with GPS devices. The remaining 14 hot spots each day were assigned to the control condition of *no patrol.* We collected data on 630 treatment units and 1260 control units by the end of the experiment.

**Fig. 2:**
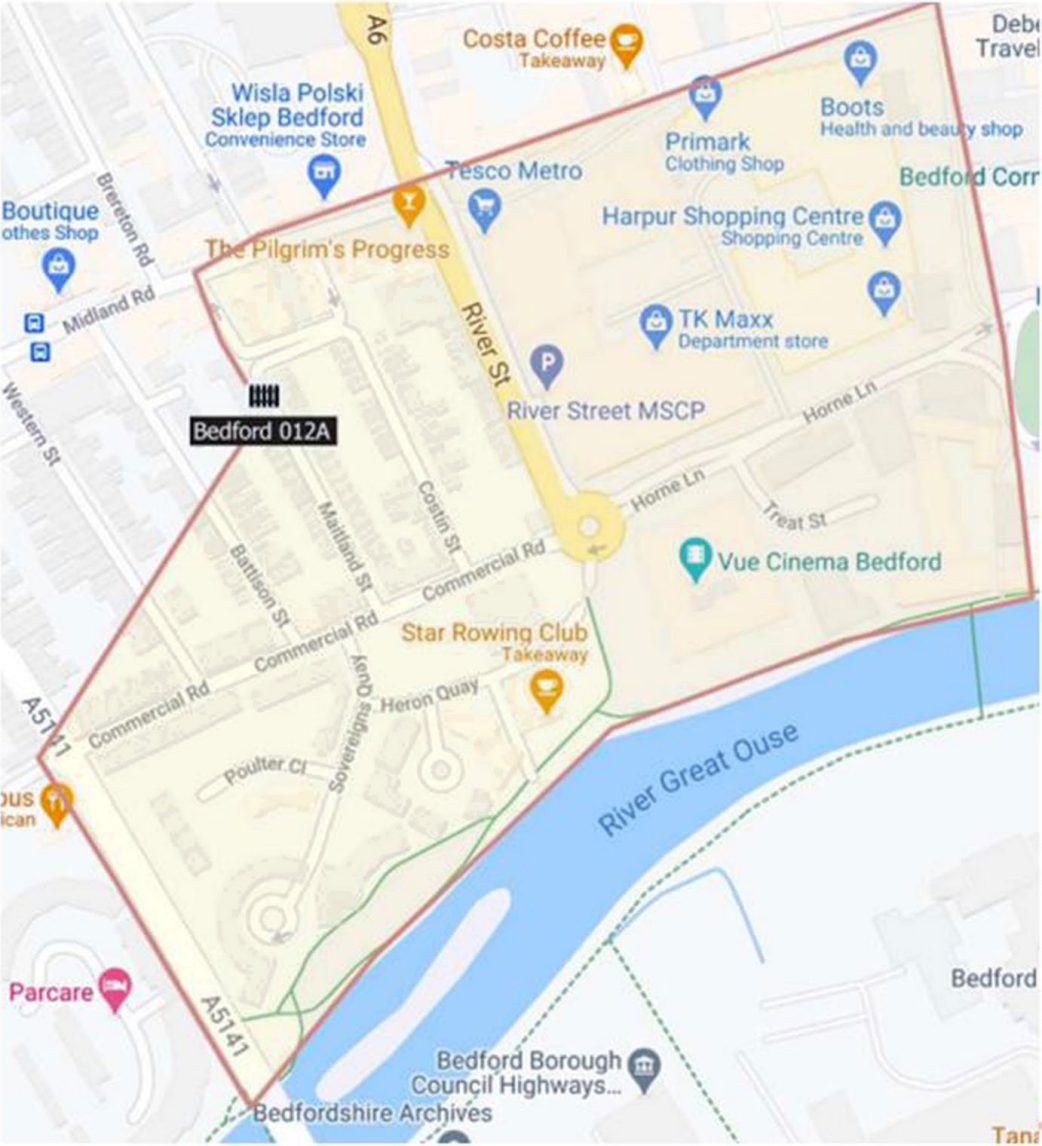
Example hot spot map provided to Operation Rowan officers

Each of the 21 LSOAs was analysed for the primary locations of non-domestic violent crimes and serious youth violence in the year before the experiment began. This analysis was used to refine the maps given to officers. An LSOA usually has about 1200 households, but they can span geographies of varying sizes. We wanted to offer officers more precision in guiding their patrols, so we isolated streets within LSOA boundaries where recorded crimes of primary interest most frequently occurred. These streets were geo-fenced using GIS software, with the mean size being 2 km^2^. After piloting the use of the GPS devices, the maps were revised manually to account for officers crossing roads and footpaths which may bisect the geofence boundaries (see Fig. 2 for example of a map provided to officers).

The hot spots were all within urban areas but split between large population towns like Luton (> 200,000 people) and smaller towns like Dunstable (35,000). Their features were commonly those of central business districts and included retail and leisure establishments and urban accommodation such as flats. Schools, train stations and police buildings were also present. Despite being drawn from towns of differing sizes, by design, Lower-layer Super Output Areas contain similar populations between 1000 and 1500 people. A full list of our 21 hot spots, their sizes and primary land use is included at Appendix. Table 3 shows the distribution of patrol and no patrol location-days among the groups of land use:

**Table 3:** Proportion of location-days assigned to patrol by land use group

Land use	Number of hot spots with this feature^a^	Total number of patrol days assigned	Proportion of location-days assigned to patrol
Central business district	7	206	33%
Out of town (various land uses including retail and office)	4	109	30%
Residential	9	277	34%

^a^This table shows 20 of the 21 hotspots. The remaining hotspot is a park and could not be grouped in any of the three categories in this table

The varying size and characteristics of our hot spots were neutralised by the repeated crossover design. Each hot spot was its own counterfactual, meaning that we could hold location-specific potential confounders constant throughout the experiment. We must acknowledge one potential limitation which did not affect ‘Sweet Spots’—the potential for spillover effects (see Ariel et al., 2021) owing to the proximity of hot spots. Nine of the 21 hot spots could be described as adjacent or sharing part of a boundary with another. In these locations, it is possible that the effect of a patrol location-day may therefore spill over into a control location-day in a neighbouring hot spot. However, we must balance this possibility with the size of our hot spots, which were larger than the normal ‘microgeographic’ definition. While Barnes et al. describe their hot spots as being at least 1 km apart, it is entirely feasible that a patrol in one of our hot spots may have been 2 km away from an adjacent hot spot which could still be classified as neighbouring territory. Ultimately, we have not analysed the locations of patrols beyond whether or not they were conducted inside the hot spot geofence, so we cannot quantitatively assess any spillover effect with precision.

A final comment to make in terms of the experimental conditions relates to the COVID-19 pandemic. This proved to be both a logistical obstacle and area of analytic opportunity. First and foremost, readers should note that restrictions on public activity were in place throughout the experimental period. At the outset of the experiment, the whole of England was in ‘lockdown,’ meaning no household mixing was permitted and no licenced premises, retail or leisure venues could open. Outdoor socialising was also restricted. These restrictions ended (for a brief period) on 2 December, during which time hospitality and retail was permitted to reopen with restrictions in place. From 19 December (40 days into the experiment), most of Bedfordshire was placed into a revised category of Tier 3, restricting indoor mixing between households in any numbers and closing all pubs, restaurants and hospitality venues. Despite being briefly relaxed on Christmas Day, these restrictions remained in place until a third national lockdown was put in place at the beginning of January 2021, which was still in place at the end of the experiment. These restrictions made it impossible to brief officers in person or observe their activities in the field. At the same time, virtual working assisted the communication of the experimental project team to continue dialogue on tracking, which we can speculate may not have happened as frequently in times when virtual meetings were less common.

Aside from logistical difficulties (and benefits), the restrictions provide a unique opportunity to analyse the effects of patrols during pandemic conditions. During the months of this trial, overall crime in Bedfordshire was down on the year prior.^4^

### Analytic Procedure

The Rowan experiment was analysed using an intention-to-treat framework, just as Barnes et al. used in ‘Sweet Spots’. Like the majority (if not totality) of patrol experiments that preceded ours, we could not entirely overcome the challenge of patrol delivery. However, the weekly GPS tracking reports enabled us to address the problem of missed patrols quickly and reasonably decisively. As Table 4 shows, we achieved compliance of better than 50% on treatment location-days and better than 97% on control location-days. This compliance level was very similar to that of the Perth experiment (Barnes et al., 2020) The treatment figures improve to more than two-thirds if we factor in patrols between 5 and 14 min.

**Table 4:** Location-day patrol delivery summary

	Treatment	Control	*p*	Effect size
Sample size (location-days)	630	1260		
*15 min* + *patrols*				
Proportion of location-days with a patrol	57.3%	2.5%	< 0.001	1.40
Mean minutes patrol per location-day	12.2	0.6	< 0.001	1.18
*Any-Duration patrols*				
Proportion of location-days with a patrol	68.4%	2.7%	< 0.001	1.62
Mean minutes patrol per location-day	13.4	0.8	< 0.001	1.32

The difference between treatment and control location-days was statistically significant and with a large magnitude of difference. These results give us a high degree of confidence in the internal validity of the findings we present in the next section. We analysed differences in the dependent variables using two-tailed independent samples *t* tests and ANOVA tests for analysis of consecutive days. We used Cohen’s *d* or *h* as the measures of effect size throughout.

To analyse displacement, we added 100 m buffers to all of our hot spots and extracted all dependent variable measurements that fell within them. We then repeated the same tests of initial deterrence as described here.

## Findings

### Initial Deterrence

The conclusions of both Braga et al. (2019) and Barnes et al. (2020) led us to expect an initial deterrence effect in crime reduction. As .

Table 5 shows, our results were broadly consistent with this expectation. The large differences in police presence in the hot spots on assigned location-days aligned with statistically significant reductions in the prevalence, volume and harm of violence and robbery and other crimes against personal victims. This equates to a 44% lower level of crime harm from serious violence in public places on days in which LSOAs were randomly assigned to one 15-min foot patrol over a day (compared to control days), as well as a 40% lower count of public crimes against personal victims.

**Table 5:** Crime and anti-social behaviour reports on treatment and control location-days

	Treatment	Control	*Sig*^*a*^	*Effect size*^*b*^
**Sample size (location-days)**	630	1260		
**Violent crime and robbery**				
Prevalence of location-days with at least one crime report	9.5%	11.0%	0.02^*^	− 0.05
Mean number of crime reports per location-day (SD)	0.11 (0.21)	0.15 (0.42)	0.01^*^	− 0.12
Mean number of CCHI days per location-day (SD)	11.6 (29.7)	20.7 (75.1)	0.00^*^	− 0.16
**Other crimes against personal victims**				
Prevalence of location-days with at least one crime report	21.7%	24.1%	0.00^*^	− 0.06
Mean number of crime reports per location-day (SD)	0.30 (0.43)	0.39 (0.78)	0.01^*^	− 0.14
Mean number of CCHI days per location-day (SD)	41.3 (118.9)	67.5 (203.4)	0.00^*^	− 0.16
**Proactively detected crimes**				
Prevalence of location-days with at least one crime report	9.3%	9.2%	1.00	0.00
Mean number of crime reports per location-day (SD)	0.12 (0.25)	0.12 (0.34)	1.00	0.00
Mean number of CCHI days per location-day (SD)	19.5 (101.5)	8.1 (85.2)	0.01^*^	+ 0.12
**Anti-social behaviour**				
Prevalence of location-days with at least one ASB report	17%	17%	1.00	0.00
Mean number of ASB reports per location-day (SD)	0.23 (0.30)	0.21 (0.27)	0.86	+ 0.07

*p < 0.05

^a^Calculated using two-tailed independent samples *t* test

^b^Calculated using Cohen’s *d* (means) or Cohen’s *h* (prevalence)

### Residual Deterrence

As Table 6 below shows, our experimental period did not have large sample sizes for high numbers of consecutive days, in comparison to Barnes et al. (2020).

**Table 6:** Available sample sizes of consecutive days’ assignment in same treatment condition

	Patrol	No patrol
1st day	427	434
2nd day	138	286
3rd day	49	183
4th day	15	118
5th day	1	80
6th day		52
7th day		33
8th day		26
9th day		15
10th day		8
11th day		5
12th day		5
13th day		4
14th day		2
15th day		2
16th day		2
17th day		2
18th day		1
19th day		1
20th day		1

Given this limitation, we collapsed the smaller frequency cells into a single group of four days and above for both the treatment and control groups. This formation of sequential assignments offers a reasonable opportunity to analyse residual deterrence. But we found no evidence, after patrol-days stopped and control days began, of a multi-day residual deterrence effect on either violence and robbery or other types of crimes against personal victims. What we did find was clear ‘cumulative’ effects from repeated patrolling.

Table 7 shows the breakdown of our crime-related outcome measures (at this point, we have dispensed with anti-social behaviour due to the lack of overall effect) for consecutive days of assignment to the treatment group.

**Table 7:** Crime outcome measure breakdown by consecutive days of assignment to the patrol condition

	First day	Second day	Third day	Fourth day + ^a^	Sig^b^
Sample size (location-days)	427	138	49	16	
**Violent crime and robbery**					
Prevalence of location-days with at least one crime report	12.3%	3.5%	6.1%	0%	0.016^*^
Mean number of crime reports per location-day (SD)	0.13 (0.37)	0.05 (0.28)	0.08 (0.34)	0 (0)	0.055
Mean number of CCHI days per location-day (SD)	14.04 (54.13)	6.68 (40.90)	8.04 (52.14)	0 (0)	0.327
**Other victim-based crimes**					
Prevalence of location-days with at least one crime report	23.2%	19.1%	20.4%	11.8%	0.510
Mean number of crime reports per location-day (SD)	0.33 (0.72)	0.27 (0.72)	0.24 (0.52)	0.12 (0.33)	0.475
Mean number of CCHI days per location-day (SD)	52.80 (231.08)	23.11 (149.67)	8.59 (52.09)	0.24 (0.66)	0.217
**Proactively detected state-based crimes**					
Prevalence of location-days with at least one crime report	8.3%	10.6%	14.3%	5.9%	0.432
Mean number of crime reports per location-day (SD)	0.11 (0.39)	0.14 (0.46)	0.22 (0.65)	0.06 (0.24)	0.324
Mean number of CCHI days per location-day (SD)	24.38 (198.33)	1.73 (7.75)	36.60 (234.89)	0.29 (1.21)	0.486

^*^p ≤ *0.05*

^a^Fourth day + actually includes only one instance of five consecutive days (as per Table 5).

^b^Calculated with ANOVA (*df* = *3629)*

Despite a lack of statistical significance, Table 7 contains several patterns of interest between its groups, which are perhaps better observed graphically.

**Fig. 3:**
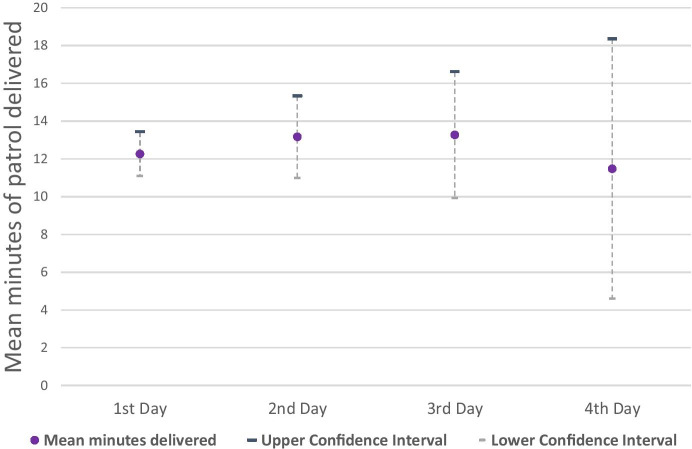
Mean minutes patrol and confidence intervals for each of consecutive days of patrol assignment

The very small sample size at day four + cannot be ignored, but at day three, the amounts of violence/robbery and other victim-based crimes were 38% and 27% lower respectively than on the first day. Harm was reduced by 43% and 76% respectively. Statistical analysis norms tell us we cannot reject the null hypothesis that there was no difference between these days, but the rigour of our design and the magnitude of the differences are too promising for police forces seeking to reduce serious crime to completely ignore. These findings are underpinned by data from the GPS units which indicate similar levels of patrol on each day (shown in Fig. 3).

As our assignment ratio was balanced towards the control group at a 2:1 ratio (see Table 5), we had greater sample sizes to analyse when it came to consecutive location-days without patrols. This is where we would have observed multi-day residual deterrence effects had they been present. 

Table 8 shows the breakdown of crime outcome measures for the first four consecutive days in which hot spots received no patrolling.

**Table 8:** Crime outcome measure breakdown by consecutive days of assignment to the no patrol condition

	First day	Second day	Third day	Fourth day + ^a^	Sig^b^
Sample size (location-days)	434	286	183	357	
**Violent crime and robbery**					
Prevalence of location-days with at least one crime report	12.0%	7.7%	15.3%	10.4%	0.099
Mean number of crime reports per location-day (SD)	0.17 (0.56)	0.09 (0.33)	0.20 (0.53)	0.15 (0.55)	0.094
Mean number of CCHI days per location-day (SD)	23.43 (98.74)	14.28 (65.12)	32.64 (137.22)	16.47 (66.57)	0.127
**Other victim-based crimes**					
Prevalence of location-days with at least one crime report	25.1%	19.6%	27.9%	24.6%	0.213
Mean number of crime reports per location-day (SD)	0.42 (0.37)	0.33 (0.44)	0.43 (0.35)	0.40 (0.45)	0.569
Mean number of CCHI days per location-day (SD)	71.45 (272.28)	58.67 (227.52)	75.60 (252.60)	65.50 (223.24)	0.873
**Proactively detected state-based crimes**					
Prevalence of location-days with at least one crime report	9.4%	7.7%	10.4%	9.8%	0.823
Mean number of crime reports per location-day (SD)	0.11 (0.37)	0.11 (0.44)	0.11 (0.35)	0.13 (0.45)	0.893
Mean number of CCHI days per location-day (SD)	19.61 (111.41)	8.57 (11.80)	1.33 (172.18)	19.57 (89.43)	0.354

^*^p ≤ *0.05*

^a^Fourth day + actually includes instances up to 20 consecutive days (as per Table 5).

^b^Calculated with ANOVA (*df* = *31,259)*

We found no consistent patterns in crime linked to consecutive days of no patrols. This is underscored by generally high *p* values from ANOVA tests throughout. Unlike Barnes et al. (2020), we found no ‘explosion’ of harm at any point. In violence/robbery and other victim-based crimes, harm was always higher on days when patrol was absent than on days when it was present, regardless of what happened in preceding days. This is robust evidence that, after each treatment day’s period of residual deterrence after the police patrol ended, there was no multi-day residual deterrence effect in Bedfordshire at all: the first day of ‘no patrol’ always had substantially more crime and more harm than the last day of ‘patrol’.

Proactively detected offending was different in that crime was at much similar levels, prevalence and harm on both treatment and control days. There appears no logical reason to explain why the most serious proactive crimes were discovered on the third consecutive day of patrol and the same consecutive day of no patrol. Here of course, we inverted our hypothesis concerning deterrence. We may expect police-discovered crimes to rise in the presence of patrols and fall in their absence. While we found a mild pattern of the former, there was no evidence of the latter.

**Fig. 4:**
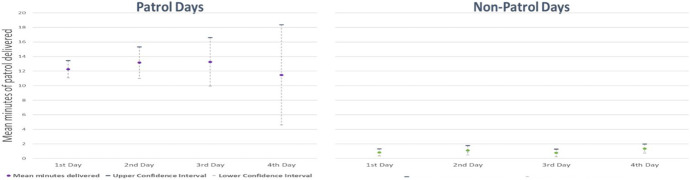
Comparison of mean patrol durations on consecutive days of patrol and no patrol assignment (with confidence intervals)

What we are confident of is that there was consistently low patrol on the consecutive days of assignment to the control condition than in the same arrangement for patrol. Figure 4 shows graphically how far apart the levels of patrol were.

### Displacement

As described in the section on procedure, we extracted all crimes meeting our eligibility criteria that fell within 100 m of the boundaries of our 21 hot spots. Where these ‘buffer zones’ intersected within an existing hot spot, we excluded the cases as this intra-hot spot displacement is already factored into the analysis of hot spot crimes. 

Table 9 shows the breakdown relating to initial (on-the-day) displacement.

**Table 9:** Crime outcome measures within 100 m of a hot spot, on both Treatment and Control days

	Treatment	Control	*Sig*^*a*^	*Effect Size*^*b*^
**Sample size (location-days)**	630	1,260		
**Violent crime and robbery**				
Prevalence of location-days with at least one crime report	5.8%	5.1%	0.74	0.03
Mean number of crime reports per location-day (SD)	0.07 (0.11)	0.05 (0.07)	0.68	0.21
Mean number of CCHI days per location-day (SD)	7.32 (12.99)	8.56 (12.02)	0.75	0.10
**Other crimes against personal victims**				
Prevalence of location-days with at least one crime report	12.6%	10.9%	0.67	0.05
Mean number of crime reports per location-day (SD)	0.14 (0.20)	0.12 (0.14)	0.75	0.12
Mean number of CCHI days per location-day (SD)	25.86 (34.15)	19.06 (18.99)	0.43	0.25
**Proactively detected state-based crimes**				
Prevalence of location-days with at least one crime report	6.1%	5.0%	0.69	0.05
Mean number of crime reports per location-day (SD)	0.06 (0.11)	0.06 (0.10)	0.89	0.00
Mean number of CCHI days per location-day (SD)	5.72 (17.80)	1.85 (7.00)	0.36	0.29

^*^p < 0.05

^a^Calculated using two-tailed independent samples *t* test

^b^Calculated using Cohen’s *d* (means) or Cohen’s *h* (prevalence)

Although the treatment location-days had higher prevalence, count and harm than the control location-days in every category, the effect sizes were small and *t* tests showed no statistical significance in any of the differences. This result seems conclusive, extra patrols did not push crimes ‘around the corner’ (at least when we describe the corner as being 100 m). However, the consecutive day assignments reveal an interesting pattern related to residual effects.

Three patterns stand out from these data. Firstly, harm in the immediate vicinity of the hot spot generally increases with every additional day of patrol (if we ignore day four on the basis of its low sample size). This is, of course, the inverse of what we found to be happening inside the hot spots. There are several reasons to be cautious with this pattern. There is no statistical significance (*p* = 0.40 for violence and robbery, *p* = 0.70 for both other victim-based crime and proactively detected crimes), and the 100-m buffer zones cover various types of terrain. However, if we are to be encouraged by the declining patterns of crime linked to consecutive days of patrol in the hot spots, then we should not completely ignore the prospect of escalation immediately outside the hot spots as the dose increases. It is also notable that harm trends towards lower levels in the surrounding areas with each day of patrol absence. This pattern is more difficult to parse at first appraisal. One might assume it indicates a return to offending within the hot spot instead of the corridor outside as offenders readjust their perceptions of risk, but that view is not supported by the picture of harm within the hot spots on those days. In the absence of statistical significance of any of these patterns, we must consider the possibility that this trend is merely statistical noise.

## Conclusions

Despite the strong evidence that hot spot policing can reduce violent crime against personal victims, police agencies remain reluctant to implement routine hot spot patrols. The co-authors of this study faced that reluctance on every day of the experiment. Yet the benefit of that work seems to be a clear reduction in serious violence and crimes against personal victims.

Having established the benefits of systematic patrolling hot spots of violent crime, the next challenge for Bedfordshire Police is to actually patrol them in a ‘business as usual’ manner. This will be no mean feat for several reasons. Firstly, as numerous prior patrol experiments have found (Ariel et al., 2016; Barnes et al., 2020; Rosenfeld et al., 2014; Sherman & Weisburd, 1995; Williams & Coupe, 2017), getting the desired level of patrol was impossible, even during the experiment. Officers routinely lacked the capacity to complete hot spot patrol assignments due to other operational demands or lack of available personnel. The level of dosage we achieved was testament to the determination and persistence of the second and third authors attending briefings and liaising with staff on the frontline. This is a well-worn path, but not one that can afford to be ignored if Bedfordshire (or any police agency) wants to achieve the potential harm reduction benefits of hot spot policing.

Whether the issue is systemic or cultural or something else was beyond the scope of our research, but this is an important gap that needs to be urgently addressed if studies such as this one are not to be the criminological equivalent of an unused vaccination. We know hot spot patrols ‘inoculate’ locations against violence; now, we must work out how to deliver a consistent programme of ‘inoculation’. There is further work to be done to understand the cost-to-benefit ratio of such a programme. Senior leaders within police forces may be tempted to replicate this study to examine effects in their own jurisdictions, and we would urge them to factor in calculation of the net cost of patrols compared to the costs saved from any crimes prevented.

Technology remains a promising delivery mechanism for the level of tracking required to operationalise our findings. The technical complexity of tracking the routes of officers is prescribed speeds, into and out of geofenced areas is not high by modern standards. However, technology alone will not solve the problem of under-delivery at hot spots. A computerised tracking system may flag the need to patrol an area before risk of serious violence escalates. But a human will always be needed to conduct the patrol, and there are many potential points of interference. Commitment to hot spot patrolling cannot simply be technological; it must also be absorbed into the tasking ‘blood flow’ of a police organisation and become part of its habits.

Like any study, ours has several limitations which readers should consider when interpreting our findings. One major limitation was our failure to secure complete compliance with our treatment and control assignments. We also highlight again that we were unable to measure the activities of all uniformed officers, only those who were assigned to Operation Rowan duty. It is highly probable that other officers operated in the hot spots during the experiment on both patrol and no-patrol days. Bedfordshire operated two regular proactive disruption operations aimed specifically at reducing serious violence, but we are confident that officers were not conducting routine foot patrols. The rest of these potential confounders we leave to our randomised design to control. Extra disruption activity took place, to be sure, but in the absence of the data required to map it, we assume that the disruption was equally distributed between the treatment and control location-days, as an unobserved confounder.

We have established that just 15 min of foot patrol in hot spot areas is sufficient to reduce both non-domestic violence and robbery and other victim-based, non-domestic crimes by around 25% in volume and 40% in harm compared to days in which patrols are not assigned to be conducted. Assigning patrols also triples the severity of crimes which are detected through police proactivity, notably drug offences. These effects are optimised not by absence, but by repeated patrols for three consecutive days. We found no robust evidence of crime being ‘pushed around the corner’, but this remains a possible effect that police forces implementing hot spot patrols can track when providing consecutive days of hot spot patrols. Non-significant displacement patterns are most likely to be statistical noise and cannot be seen as a reason *not* to conduct hot spot patrols. In the end, the evidence *supporting* hot spot patrols increases daily—this study included.

## Appendix

**Table:**

							Violence & robbery			Other victim crimes	
Hot spots code	Size km sq	Primary land use	Days assigned to patrol	Days patrol delivered	Percent assigned days delivered	Mean minutes per patrol day	Net diff prev	Net diff freq	Net diff harm	Net diff prev	Net diff freq
Bedford 009A	2.85	Public park	38	25	65.8%	25.4	3%	0.03	− 28.01	− 10%	− 0.13
Bedford 010A	1.76	Central business district	27	19	70.4%	20.3	1%	− 0.14	− 26.53	19%	0.30
Bedford 012A	1.48	Central business district	25	15	60.0%	19.5	4%	0.01	8.80	− 13%	− 0.19
Bedford 012D	1.74	Central business district	31	19	61.3%	20.6	− 4%	− 0.04	− 6.16	− 10%	0.16
Bedford 012F	1.75	Central business district	36	23	63.9%	17.5	− 5%	− 0.05	− 18.09	− 3%	− 0.06
Bedford 014E	2.08	Out of town offices, retail	28	13	46.4%	23.4	10%	0.10	18.46	2%	0.04
Bedford 015B	1.69	Out of town offices, retail, car parking	28	16	57.1%	18.1	− 3%	− 0.03	6.36	− 1%	0.03
Bedford 015C	1.34	Residential	25	18	72.0%	20.1	1%	0.01	− 5.16	− 4%	− 0.13
Bedford 018C	1.88	Residential	34	22	64.7%	23.0	5%	0.05	6.27	8%	− 0.02
Bedford 021A	1.29	Central business district	29	14	48.3%	27.3	− 4%	− 0.04	− 2.12	− 8%	− 0.21
Bedford 029C	3.02	Central business district	34	17	50.0%	20.2	9%	0.09	11.24	− 3%	− 0.05
C Beds 028F	1.88	Residential	28	18	64.3%	26.6	− 4%	− 0.04	− 6.96	− 2%	− 0.04
Luton 003C	1.57	Residential	41	30	73.2%	20.0	2%	0.02	8.09	2%	0.01
Luton 003D	1.65	Residential, school	19	13	68.4%	17.9	− 2%	− 0.02	− 0.03	− 5%	− 0.10
Luton 005A	2.06	Residential	25	24	96.0%	20.5	− 1%	− 0.01	− 0.77	4%	0.04
Luton 009C	1.91	Residential	30	26	86.7%	22.0	0%	-	-	− 6%	− 0.06
Luton 009E	2.45	Residential, school	38	30	78.9%	19.3	10%	0.10	6.96	2%	− 0.02
Luton 009F	2.29	Residential, school	37	31	83.8%	20.5	3%	0.03	− 0.45	16%	0.30
Luton 018E	1.59	Out of town retail, park, residential	32	24	75.0%	17.9	− 6%	− 0.06	2.57	− 9%	− 0.52
Luton 018F	2.99	Major central business district, rail	24	19	79.2%	21.4	2%	0.24	15.68	7%	− 0.04
Luton 021A	1.98	Out of town retail, residential	21	14	66.7%	23.6	5%	0.06	44.14	0%	-

## Footnotes

1 Anti-social behaviour is an action which lacks consideration for other but does not meet the definition of a criminal offence. Examples include noise disturbances or rowdy behaviour.

2 LSOA is a geographical hierarchical unit for English and Wales used by the Office for National Statistics to report on local statistics.

3 HMP Bedford is situated in one of the identified hot spots.

4 Overall crime figures collated for us by Bedfordshire’s Performance Analysts indicated a 12 to 17% reduction. This figure is crude, because it includes all crime types and does not differentiate between place or method of discovery.
